# Experimental Demonstration of Four-Dimensional Photonic Spatial Entanglement between Multi-core Optical Fibres

**DOI:** 10.1038/s41598-017-04444-8

**Published:** 2017-06-27

**Authors:** Hee Jung Lee, Sang-Kyung Choi, Hee Su Park

**Affiliations:** Korea Research Institute of Standards and Science, Daejeon, 34113 South Korea

## Abstract

Fibre transport of multi-dimensional photonic quantum states promises high information capacity per photon without space restriction. This work experimentally demonstrates transmission of spatial ququarts through multi-core optical fibres and measurement of the entanglement between two fibres with quantum state analyzers, each composed of a spatial light modulator and a single-mode fibre. Quantum state tomography reconstructs the four-dimension entangled state that verifies the nonlocality through concurrences in two-dimensional subspaces, a lower bound of four-dimensional concurrence and a Bell-type CGLMP inequality.

## Introduction

The advantages in using single photons as quantum information carriers are the readily accessible infinite dimensionality of their quantum states and their robustness over long-distance transmission. High-dimensional encoding on single photons offers the potential to enhance quantum information processing in various ways. The dimensions added to simple qubits can increase the data rate^1^, lower the acceptable error rate for secure quantum communications^2^, and simplify quantum logic circuits^3^. Spatial modes^4–9^, temporal modes^10, 11^ or colours^12^ of photons can carry such multi-dimensional quantum states, namely qu*d*its. Spatial modes such as optical paths and orbital angular momentum states provide an advantage of relative ease in controlling arbitrary superpositions of multiple logical states |1〉, |2〉, …, |*d*〉 (*d* > 2) compared to other types of qu*d*its, and have been successfully applied to protocols relying on high-dimensional quantum state measurements such as hyper-entanglement-based quantum communication and quantum process tomography^13, 14^.

To avoid decoherence between the spatial modes due to ambient vibration noise, a practical implementation usually consists of paraxial modes sharing common holograms for state control^4^ or inherently stable structures using beam displacing prisms and Sagnac interferometers^3, 15, 16^. This work shows that an optical fibre with multiple cores can also transport high-dimensional spatial quantum states, by which space restriction and vulnerability to weather condition can be overcome. Fibre transport of spatially entangled qubits (*d* = 2) through a few-mode fibre has been demonstrated^17, 18^, and we extend the work to four-dimensional entanglement based on a scheme that ideally can transmit large-*d* entanglement without inter-modal decoherence.

Multi-core fibres (MCFs) have been developed for high-power fibre laser amplifiers based on phase-locked beam combinations^19^ and for space-division-multiplexing optical communications^20, 21^. Quantum communication through an MCF line based on a faint laser source has recently been reported^22, 23^. This work employs a commercially available four-core fibre to guide spatially-entangled telecom-wavelength photon pairs produced by spontaneous parametric down-conversion (SPDC). After transmission through the MCFs, each ququart (*d* = 4) is analyzed through a spatial light modulator (SLM) and spatial-mode filtering by a single-mode fibre (SMF). The two-ququart entanglement is verified by two-qubit concurrences in six pairwise two-mode subspaces, a lower bound of multi-dimensional bipartite concurrence^24^ and a generalized Bell inequality for high dimensions^25^, calculated from the result of quantum state tomography composed of 256 projection measurements.

## Results

### Preparation of entangled photon pairs in MCFs

**Figure 1:**
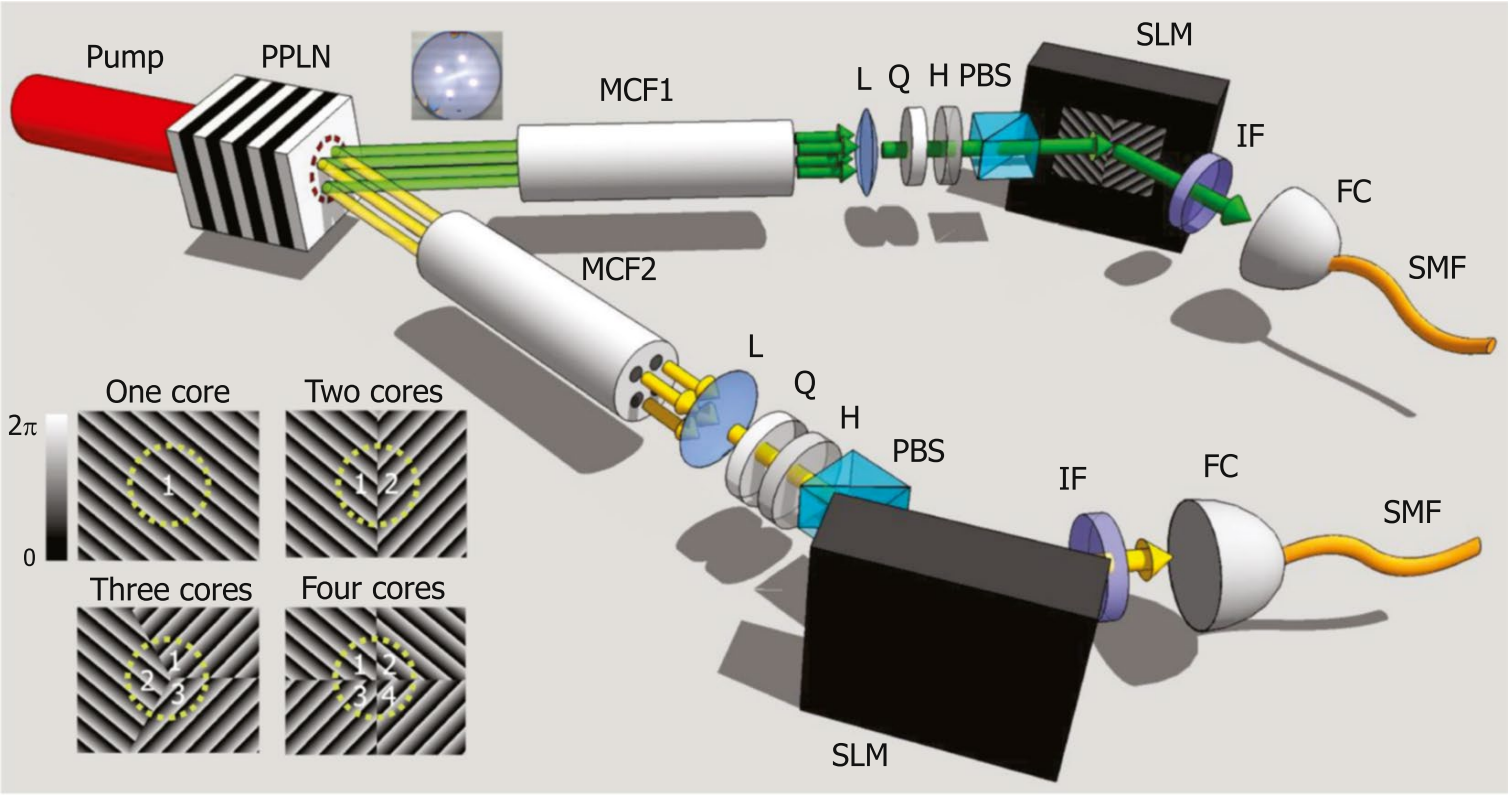
Schematic of the experimental setup. Photon pairs are produced by non-collinear degenerate type-0 SPDC pumped by a continuous-wave laser beam with wavelength of 780 nm. (Imaging lenses between the PPLN crystal and the MCFs are omitted.) Insets: MCF cross-section and phase patterns on the SLM. PPLN: periodically poled lithium niobate; MCF: multi-core fibre; L: lens; Q: quarter-wave plate; H: half-wave plate; PBS: polarizing beam splitter; SLM: spatial light modulator; IF: interference filter; FC: fibre coupler; SMF: single-mode fibre.

With our experimental setup (Fig. 1), spatially entangled photon pairs are generated by non-collinear degenerate type-0 SPDC in a periodically poled lithium niobate (PPLN) crystal, pumped by a continuous-wave diode laser (wavelength 780 nm). The down-converted photon pairs with a centre wavelength of 1560 nm propagate with a divergence angle of 3.1°. Each photon is respectively coupled to an MCF (Fibrecore SM-4C 1500, total length 30 cm) that has four identical single-mode cores (mode field diameter 8 *μ*m, NA 0.14–0.17) at the vertices of a 37 *μ*m × 37 *μ*m square. The photon-collecting end faces of the two MCFs are imaged onto the identical position of the PPLN crystal within the pump beam spot.

We post-select the photon pairs coupled to the cores of the two MCFs. Because the distances between the core images are greater than the transverse shift of photons inside the PPLN crystal, only four combinations between the identical-index cores *i* = 1, 2, 3, 4 of both MCF1 and MCF2 yield non-zero overlap integrals that lead to photon pair generation^26^. The post-selected quantum state propagating through the MCFs can be expressed as a four-dimensional entangled state:

1 $$|{\rm{\Psi }}\rangle =a{|1\rangle }_{1}{|1\rangle }_{2}+b{|2\rangle }_{1}{|2\rangle }_{2}+c{|3\rangle }_{1}{|3\rangle }_{2}+d{|4\rangle }_{1}{|4\rangle }_{2},$$

where |*i*〉_*j*_ denotes the single-photon state in core *i* of MCF *j*, and *a*, *b*, *c* and *d* are complex constants determined by the pump beam profile and the imaging configuration. The initially vertically polarized photons undergo birefringence in the MCFs because of their curvature and intrinsic stress. The output polarizations differ between the cores in our experiment; therefore, we convert the ‘average’ polarization to horizontal polarization using a quarter-wave plate and a half-wave plate after collimation^27^. The vertical-polarization component is filtered out by a polarizing beam splitter. This core dependence of the birefringence can in principle be suppressed using polarization-maintaining MCFs^28, 29^. The polarization-filtered photons are reflected by an SLM before being narrow-band-filtered with an interference filter (half-maximum bandwidth 8.3 nm) and detected by an SMF-coupled avalanche photodiode single-photon counter. The phase patterns on the SLMs (inset of Fig. 1) determine the spatial quantum state measured by the single-photon counters.

### State projection with the SLM

The SLM is a reflection-mode phase modulator with a 792 × 600 array of 20 *μ*m × 20 *μ*m pixels (Hamamatsu LCOS-SLM). The grating-like patterns in Fig. 1 control the reflection angle of the incident beam by means of the direction and magnitude of the gradient of the linear phase. The pattern becomes a saw-tooth function because of the phase reset between 2*π* and 0. The SLM surface is divided into subsections that connect different cores to the output SMF when the superposition states of multiple core modes are measured^30^. The images of the MCF and the SMF are centred at the SLM patterns (Fig. 1) to equalize the coupling efficiencies of the four MCF cores to the output SMF following the procedures described in Ref. 30. Coupling to *N*-core states reduces the total coupling efficiency to 1/*N* in our scheme; for example, projection of the output state to |*ψ*_*out*_〉 to $|{\psi }_{meas}\rangle =(|1\rangle +\cdots +|N\rangle )/\sqrt{N}$ results in coincidence counts proportional to |〈*ψ*_*meas*_|*ψ*_*out*_〉|^2^/*N*. To avoid the reduction of the detection efficiency in practical applications of such large-*N* states, optical interferometric circuits for unitary transformation of the spatial modes will have to be developed possibly utilizing integrated-optic devices^31^. The coupling efficiencies from one core of the MCF to the output SMF are 100%, 25%, 11% and 6.3% in an ideal case, and 54%, 13%, 4.8% and 3.6% in our experiments for one-, two-, three- and four-core superposition states, respectively. The differences are attributed to the reflectivity (90%) of the SLMs and the efficiency of the mode coupling by two aspheric lenses (focal length 15 mm) between the MCF and the SMF.

### Correlations between core modes and superposition states

**Figure 2:**
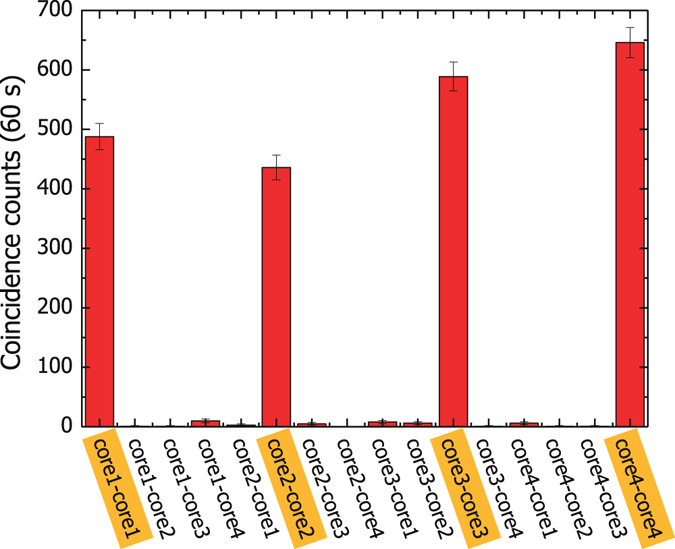
Correlations between the core modes (core *i*-core *j*: projection to core *i* of MCF1 and core *j* of MCF2).

We first measure the spatial correlation in Equation (1) by the coincidence counts between the core modes (Fig. 2). The unwanted correlations, i.e. all the correlations except the four dominant ones, sum to 2.0 ± 0.3% of the total of all counts. The error is the statistical uncertainty corresponding to $\pm \sqrt{\,{\rm{counts}}}$ of the coincidence counts. Accidental coincidence counts were negligible in our experimental conditions.

**Figure 3:**
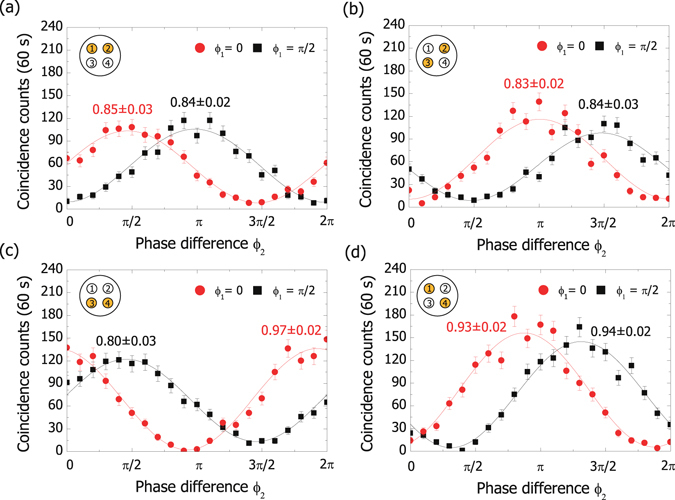
Correlations between two-core superposition states. Photons 1 and 2 are projected onto the states $({|i\rangle }_{1}+{e}^{i{\varphi }_{1}}{|j\rangle }_{1})/\sqrt{2}$ and $({|i\rangle }_{2}+{e}^{i{\varphi }_{2}}{|j\rangle }_{2})/\sqrt{2}$, respectively. *ϕ*_1_ is fixed to 0 (circles) or *π*/2 (squares). *ϕ*_2_ is scanned from 0 to 2*π*. The numbers are the fringe visibilities of the data. (**a**) (*i*, *j*) = (1, 2), (**b**) (*i*, *j*) = (2, 3), (**c**) (*i*, *j*) = (3, 4), (**d**) (*i*, *j*) = (4, 1).

Correlations between two-core superposition states verify the coherence between the four states in Equation (1) (Fig. 3). Patterns with two subsections are loaded on the SLM, where the relative phase *ϕ*_*i*_ (*i* = 1, 2) is adjusted by shifting the pattern of one subsection along the direction of the phase gradient. The results clearly show interference fringes with an average visibility of 0.88 ± 0.02. The phase biases of the interference fringes are attributed to a slight difference in refractive index between the cores of the MCF.

The sources of departure from the ideal visibility are under investigation. Core selectivity of the measurement setup does not significantly compromise the visibility because classical-light Mach-Zehnder interferometers using the same type of MCF and SLMs achieve visibilities greater than 99%^30^. Interference with the unwanted correlation components in Fig. 2 degrades the visibility by 0.02 on average. Non-uniformity of the four major components in Fig. 2 reduces the visibility by 0.01. Group refractive index mismatch between the cores of the MCF (within 6.5 × 10^−4^)^30^ can also reduce the visibility when photons has finite wavelength bandwidth (8.3 nm) and the two MCFs has a length mismatch. Considering the uncertainty (<1 cm) in the fibre length measurement, the visibility reduction due to the last factor is <0.003. Remaining error sources cause the unexplained amount of decoherence between the terms in Equation (1). Our non-collinear geometry can introduce slight temporal distinguishability between core modes by core-dependent path-length difference between the two MCFs. Non-uniformity of the MCF can also increase the decoherence due to the group index mismatch.

### Geometric alignment of the MCF cores

**Figure 4:**
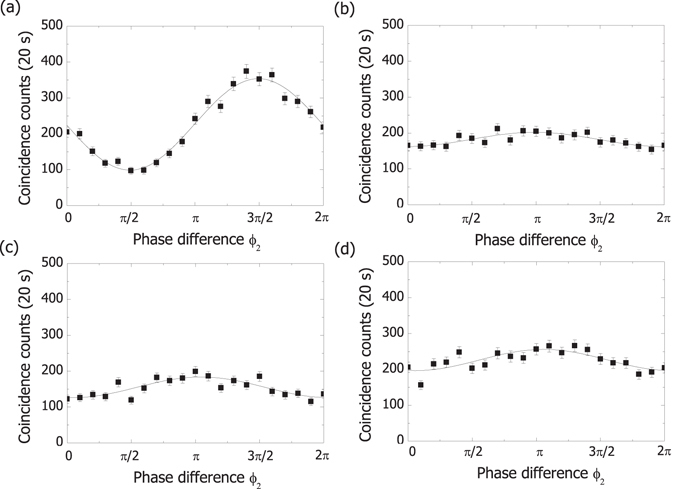
Interference fringes with varying alignment of the four cores: Coincidence counts with photons 1 and 2 projected onto $({|1\rangle }_{1}+{|4\rangle }_{1})/\sqrt{2}$ and $({|1\rangle }_{2}+{e}^{i{\varphi }_{2}}{|4\rangle }_{2})/\sqrt{2}$, respectively. MCF1 is rotated with respect to the fibre axis by (**a**) 0°, (**b**) 90°, (**c**) 180° and (**d**) 270°. The interference filters in Fig. 1 are removed for these measurements.

The interference fringe visibilities in Fig. 3 are a good measure for geometric positioning of the MCF cores. Because the cores in the MCFs are not perfectly identical, the same cores of MCF1 and MCF2 need to overlap to suppress the decoherence caused by the temporal distinguishability after propagation through the MCFs. With proper alignment, the coherence between the different core modes in Equation (1) is maintained if the lengths of the fibres are the same and the differential time delay between the cores is smaller than the coherence time (>1 *μ*s) of the pump laser. Otherwise maintaining the coherence of the entangled state requires the differential group delay to be smaller than the coherence time of the photons, that is, much shorter than ~400 fs. Figure 4 shows the correlation between superposition states of two diagonal cores with MCF1 rotated by 0°, 90°, 180° and 270° and all other optical components fixed. We removed the interference filters during these measurements to better clarify the optimal position of the cores [Fig. 4(a)]. The non-ideal maximum visibility of 0.56 ± 0.02 may have resulted from a fibre length mismatch of <1 cm between MCF1 and MCF2 when considering the group index difference of 6.5 × 10^−4^ between cores 1 and 4 and a half-maximum wavelength bandwidth greater than 150 nm for the photons.

### Quantum state tomography and entanglement measures

**Figure 5:**
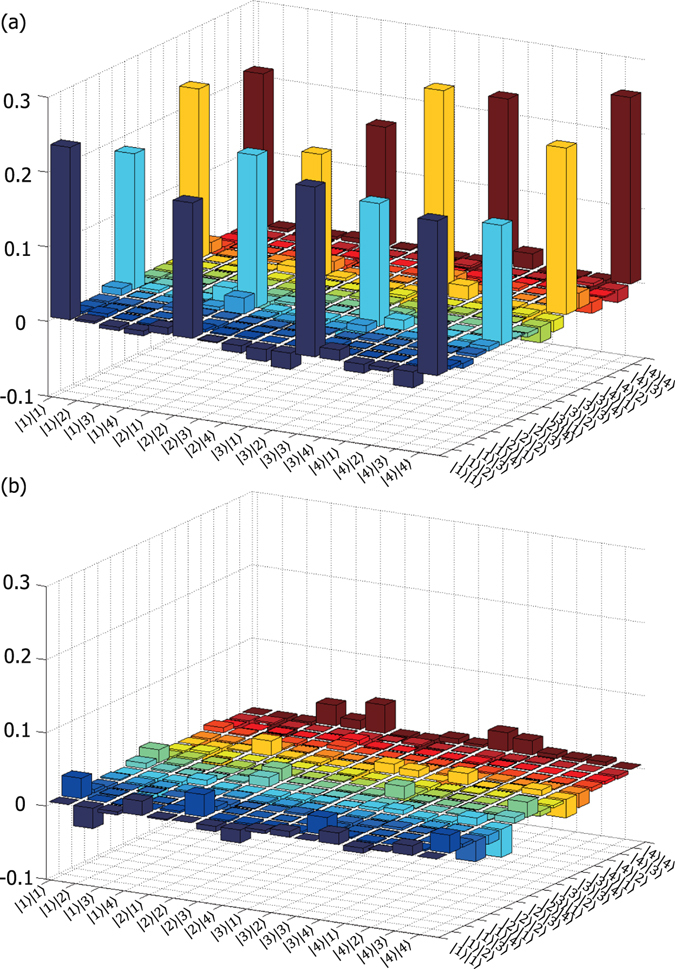
Two-ququart density matrix $\hat{\rho }$ reconstructed by quantum state tomography: (**a**) real and (**b**) imaginary parts.

Quantum state tomography reconstructs the density matrix (Fig. 5) of the output state from 256 = 16 × 16 projection measurements composed of four one-core states and twelve two-core superposition states $((|1\rangle +|2\rangle )/\sqrt{2})$, $((|1\rangle +i|2\rangle )/\sqrt{2})$, etc.) of each photon. Each projection measurement was coincidence counts for 60 s. To better compare the result with the symmetric maximally entangled state |*β*〉 = 1/2(|1〉|1〉 + |2〉|2〉 + |3〉|3〉 + |4〉|4〉), we re-phased the reconstructed state ${\hat{\rho }}_{0}$ by adding phases 0, *ϕ*_2_, *ϕ*_3_ and *ϕ*_4_ to the |1〉, |2〉, |3〉 and |4〉 states, respectively, of photon 1. Here, ${\varphi }_{{\rm{2}}}=\langle 1|\langle 1|{\hat{\rho }}_{0}|2\rangle |2\rangle$, ${\varphi }_{3}=\langle 1|\langle 1|{\hat{\rho }}_{0}|3\rangle |3\rangle$ and ${\varphi }_{4}=\langle 1|\langle 1|{\hat{\rho }}_{0}|4\rangle |4\rangle$. Hence the final density matrix $\hat{\rho }$ (Fig. 5) is given by $\hat{\rho }=U{\hat{\rho }}_{0}{U}^{\dagger }$ (*U* = *M* ⊗ *I*), where *M* is the phase shift operator and *I* is the identity operator. Ideally the sixteen peaks in Fig. 5(a) should be constant at 0.25 and the other elements in Fig. 5(a) and (b) should be zero.

The Schmidt number of the reconstructed state is 3.79 ± 0.03, which verifies the dimensionality of the state. The fidelity ${\rm{Tr}}\{\sqrt{{\hat{\rho }}^{\mathrm{1/2}}|\beta \rangle \langle \beta |{\hat{\rho }}^{\mathrm{1/2}}}\}$ with the ideal maximally entangled state is 0.91 ± 0.01, and the state purity ${\rm{Tr}}\{{\hat{\rho }}^{2}\}$ is 0.73 ± 0.03. To quantify the quantum coherence between the components, we calculate the two-qubit concurrences of the six two-dimensional subspaces (Table 1).

**Table 1:** Two-qubit concurrences in the two-dimensional subspaces.

Ideal State	Experimental Concurrence
$1/\sqrt{2}({|1\rangle }_{1}{|1\rangle }_{2}+{|2\rangle }_{1}{|2\rangle }_{2})$	0.82 ± 0.05
$1/\sqrt{2}({|1\rangle }_{1}{|1\rangle }_{2}+{|3\rangle }_{1}{|3\rangle }_{2})$	0.89 ± 0.04
$1/\sqrt{2}({|1\rangle }_{1}{|1\rangle }_{2}+{|4\rangle }_{1}{|4\rangle }_{2})$	0.85 ± 0.03
$1/\sqrt{2}({|2\rangle }_{1}{|2\rangle }_{2}+{|3\rangle }_{1}{|3\rangle }_{2})$	0.67 ± 0.06
$1/\sqrt{2}({|2\rangle }_{1}{|2\rangle }_{2}+{|4\rangle }_{1}{|4\rangle }_{2})$	0.72 ± 0.06
$1/\sqrt{2}({|3\rangle }_{1}{|3\rangle }_{2}+{|4\rangle }_{1}{|4\rangle }_{2})$	0.85 ± 0.04

As an entanglement measure, we calculate a lower bound of concurrence C(*ρ*) defined for multi-dimensional bipartite states. C(*ρ*) is bounded below by the *quasipure* lower bound C_*qp*_^24^:

2 $${\rm{C}}(\rho )\ge {{\rm{C}}}_{qp}(\rho )=\,{\rm{\max }}(\mathrm{0,}\,{S}_{0}-\sum _{i > 0}{S}_{i})\mathrm{\ ,}$$

where *S*_*i*_’s are the singular values of a matrix with elements ${{\rm{T}}}_{ij}=\sqrt{{\mu }_{i}{\mu }_{j}}\langle {{\rm{\Psi }}}_{i}|\otimes \langle {{\rm{\Psi }}}_{j}|\chi \rangle$. The parameters are constructed with the spectral decomposition *ρ* = ∑_*i*_ *μ*_*i*_|Ψ_*i*_〉〈Ψ_*i*_|, *|χ*〉 ∝ A|Ψ_0_〉⊗|Ψ_0_〉, and a projector-valued operator A defined as:

3 $$A=4\sum _{i < j,k < l}(|ikjl\rangle -|jkil\rangle -|iljk\rangle +|jlik\rangle )\times ({\rm{H}}{\rm{.c}}{\rm{.}}),$$

where *i* and *j* (*k* and *l*) enumerate the basis vectors of the first (second) partition^24^. The C_*qp*_ values for the ideal maximally entangled state in Equation (1) (*a* = *b* = *c* = *d*) and the three-dimensionally entangled state (*a* = *b* = *c*, *d* = 0) are 0.61 and 0.41, respectively. The quantum state tomography results shown in Fig. 5 yield C(*ρ*) ≥ C_*qp*_(*ρ*) = 0.47 ± 0.02, and verify the four-dimensional entanglement.

We further test the generalized Bell-type CGLMP inequality for a high-dimensional two-photon quantum state^25, 32^ with the reconstructed state. The Bell parameter *I*_*d*_ (*d* ≥ 2) fulfils the inequality *I*_*d*_ ≤ 2 by local variable theories, and reaches its maximum of *I*_4_ = 2.9727 with a non-maximally entangled state^32^. The symmetric maximally entangled state theoretically yields a value of *I*_4_ = 2.8962. We use the general Bell operator matrix form^32^ to calculate *I*_4_ from the density matrix (Fig. 5), which leads to *I*_4_ = 2.27 ± 0.06 violating the inequality by 4.5 standard deviations. Other entanglement quantifiers such as a G-concurrence^33^ will also be calculable from the reconstructed density matrix. Direct measurement of observables detecting the nonlocality such as *I*_*d*_ will require extension of our projection measurements to more-than-two-core superposition states with further characterization of the amplitude and phase responses of the measurement setup.

## Discussion

**Figure 6:**
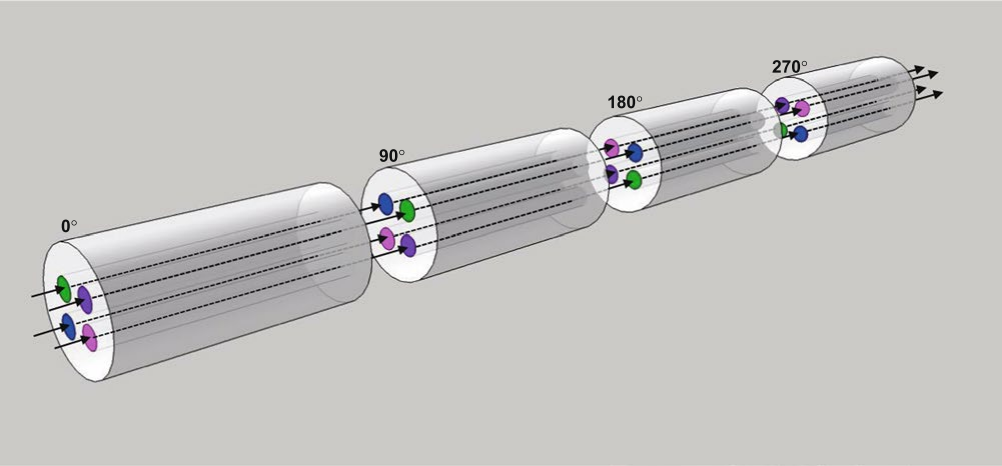
Compensation of the decoherence due to group delay mismatch between the cores. Four equal-length fibres are spliced such that cores 1, 2, 3 and 4 are respectively coupled to cores 2, 3, 4 and 1 at the three splice points. The numbers are the relative azimuthal angles of the fibres.

We discuss an extension of our scheme to general entangled states and to longer-distance transmission. Coefficients *a*, *b*, *c* and *d* in Equation (1) depend on the spatial profile of the pump beam. The same technique using an SLM to control the photonic spatial mode is also applicable to the pump laser to realize an arbitrary combination of the coefficients. Quantum information processing based on hyper-entanglement or hybrid entanglement can be realized if one incorporates qubit-joining or qubit-transduction techniques that can convert multiple qubits into a single spatial qu*d*it^34–36^. Ideal MCFs can transport arbitrary quantum states over long distances without suffering decoherence. However, slight differences in group refractive indices between the cores of a practical MCF limit the transmission distance, which is less than a few meters considering the wavelength bandwidth (8 nm) of photons and the group index differences (≳10^−4^)^30^ between the cores of the MCF in our experiments. This limit can be overcome by placing mode converters that cyclically permutate the cores at each of the *d* − 1 uniformly distributed points, where photons propagating through cores 1, 2, … and *d* are respectively coupled to cores 2, 3, …, *d* and 1 as shown in Fig. 6. When the cores are located on the circumference of a circle as in Fig. 1, this mode converter can be realized by splicing two MCF sections with a relative angle detuning of 360/*d*°.

In summary, we have proven the experimental feasibility of transmission of four-dimensional spatially entangled photon pairs through commercially available multi-core optical fibres. Quantum state tomography composed of one-core states and two-core superposition states has quantitatively verified the non-classicality of the transmitted photonic states through the concurrences of six two-dimensional subspaces, the lower bound of four-dimensional concurrence and the violation of the four-dimensional CGLMP inequality. These results suggest the usefulness of optical fibres for multi-dimensional quantum communications and quantum interfaces.

## Methods

### Photon pair generation in the PPLN crystal

The PPLN crystal (Covesion MSHG1550) used in this work has a poling period of 19.5 *μ*m and a length of 1 mm. The pump laser (Toptica DL100) for SPDC is a Littrow-type external-cavity diode laser that produces continuous-wave output with optical output power of 67 mW and linewidth of <1 MHz. The 1/*e* ^2^ spot diameter of the pump beam on the PPLN crystal surface is 415 *μ*m. The end faces of the MCFs are five-fold magnified onto the PPLN surface, therefore the four core images with mode field diameter of 40 *μ*m are located at the vertices of a 185 *μ*m × 185 *μ*m square, which are well within the pump beam spot. The heralding efficiency, the ratio between the coincidence counts and the single counts, for the four core *i*-core *i* (*i* = 1, 2, 3, 4) correlations in Fig. 2 is about 7% after compensating for the detector efficiency.

### Quantum state tomography

The quantum state tomography is composed of projection measurements of 256 separable states of the form |*ψ*_1_〉|*ψ*_2_〉. When one or two of the states |*ψ*_1_〉 and |*ψ*_2_〉 is a two-core superposition state, we multiply the coincidence counts by two or four, respectively, before applying the data to maximal likelihood estimation (performed by lsqnonlin function of Matlab) considering the reduction of the coupling efficiency to the output lead SMFs. While the 12 two-core superposition state projection of each photon, there are cases that an MCF core is coupled to the output SMF after reflected by different subsections of the SLM. We measure the phase bias between the two cases by measuring the interference of the two beams from a single MCF core via the two subsections. The measured bias is within 5% of the wavelength for all the combinations, and compensated for during the phase-sensitive measurements.
